# Self-sustained GaIn droplet oscillations by coupled electrochemical and mechanical driving forces

**DOI:** 10.1039/d6sm00708b

**Published:** 2026-09-29

**Authors:** Yi Li, David Paulovics, Mathieu Odijk, Javier Rodríguez Rodríguez, Ivana Qianqi Lin, Detlef Lohse, Christian A. Nijhuis

**Affiliations:** a Hybrid Materials for Opto-Electronics Group, Department of Molecules and Materials, MESA+ Institute for Nanotechnology and Center for Brain-Inspired Nano Systems, Faculty of Science and Technology, University of Twente P.O. Box 217 7500 AE Enschede The Netherlands c.a.nijhuis@utwente.nl; b Physics of Fluids Group, Max Planck Center for Complex Fluid Dynamics, and J.M. Burgers Centre for Fluid Dynamics, University of Twente P.O. Box 217 7500AE Enschede The Netherlands; c Integrated Devices and Systems Group, MESA+ Institute, University of Twente P.O. Box 217 7500 AE Enschede The Netherlands; d Department of Thermal and Fluids Engineering, “Gregorio Millán Barbany” Institute, Universidad Carlos III de Madrid Spain; e Max Planck Institute for Dynamics and Self-Organization Am Faßberg 17 37077 Göttingen Germany

## Abstract

Self-sustained oscillations of liquid-metal droplets represent a striking example of electrochemical–mechanical coupling in active matter. These liquid-metal droplets immersed in an aqueous solution oscillate toward and away from an electrode, in response to a continuous applied voltage. Recently, these oscillations have been studied in gallium-indium (GaIn) droplets. Though it is understood that the GaIn droplet self-oscillations are driven by surface tension variations, the details of the underlying mechanism remain incompletely understood, in part because previous investigations have relied primarily on optical observations. Here, we introduce an experimental platform that enables simultaneous electrochemical and optical characterization of self-oscillating GaIn droplets in an aqueous NaOH electrolyte under wet-electrochemical conditions in both an unconfined and laterally confined (Hele–Shaw) geometry. By correlating the time dependence of the current with the droplet dynamics, we could identify five distinct stages in the oscillation cycle, involving (i) droplet-electrode contact and positive charging, (ii) droplet spreading due to oxide growth, (iii) droplet detachment and asymmetrical contraction, (iv) inertial movement away from the electrode, and (v) electrowetting-driven return to the electrode. The measurements reveal two sequential driving mechanisms, namely asymmetrical contraction-induced inertial movement (following oxide dissolution) and an electrowetting-induced Marangoni flow associated with charge redistribution at the liquid-metal/electrolyte interface. Furthermore, we observe reversal of the interfacial flow direction and transient decoupling of surface and bulk motion. These findings provide new insight into the electrochemical–mechanical driving mechanism governing liquid-metal droplet oscillators in aqueous solutions.

## Abstract 2

Self-sustained oscillations arise from coupled electrochemical and mechanical processes in GaIn-droplet systems. Asymmetric contraction and electrowetting-induced Marangoni flow sequentially drive the droplet away from and back toward the electrode.

## Graphical abstract

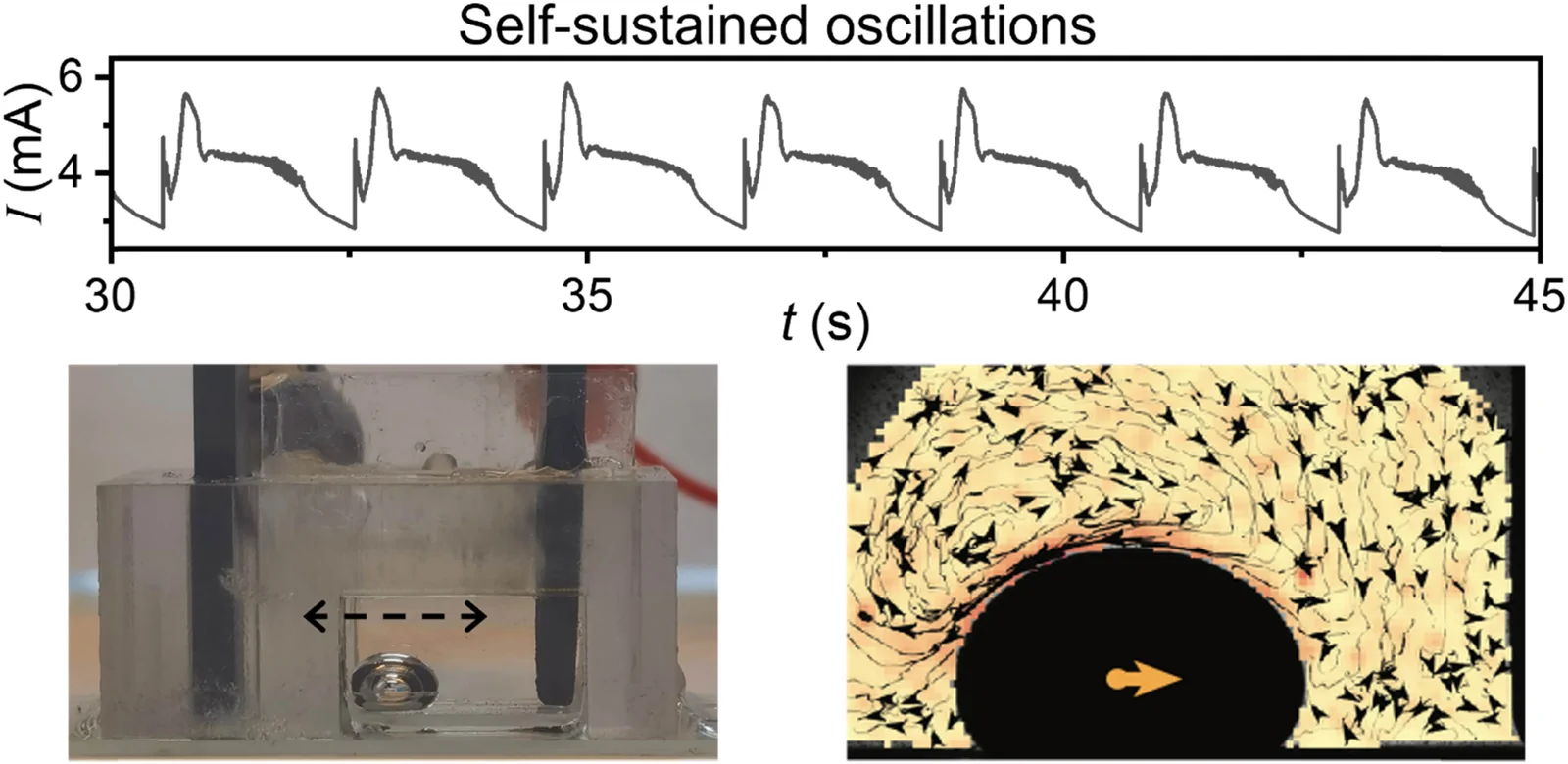

## 1. Introduction

Oscillatory droplets represent an intriguing class of active soft-matter systems in which chemical, electrical, and mechanical processes are coupled to generate spontaneous periodic motion.^1,2^ Such systems are far from equilibrium,^3^ transport energy from large to small scale,^4^ self-organize^5^ and display locomotion.^6^ While extensive research has been devoted to chemically driven and motile droplets,^7,8^ liquid-metal droplets are of importance in this context. A well-known example is the “beating heart” oscillatory system based on Hg droplets,^9,10^ which in recent years have been replaced with non-toxic GaIn droplets.^11,12^ The liquid-metal droplets offer unique opportunities owing to their exceptionally high electrical conductivity, large interfacial tension, and the presence of a dynamic oxide layer that can be reversibly modified by electrochemical reactions.^13–15^ These properties enable rich electrohydrodynamic behaviors that are not accessible in conventional oil or aqueous droplets, opening the door to new applications in soft-robotics,^16,17^ signal conversion,^18,19^ microfluidic pumping,^20^ or additive manufacturing.^21,22^ In alkaline electrolytes, liquid-metal droplets subjected to electric fields can exhibit sustained oscillatory motion arising from complex processes including charge transfer, oxidation, surface tension variations, and droplet deformation.^18,23^ To date, most studies in liquid-metal droplet oscillators have focused on visual characterization of the droplet motion (using high speed cameras for example), while the accompanying electrical response has received comparatively little attention.^18,24–26^ As a result, the dynamic coupling between the electrochemical processes and the mechanical response of the droplet remains incompletely understood. Here, by combining real-time optical imaging, of both the droplet motion and the flow around it, with simultaneous measurements of the electrical current, we directly correlate the electrochemical and mechanical signatures of the oscillation cycle, providing new insights into the mechanism governing liquid-metal droplet oscillators.

In general, liquid-metal droplets oscillate due to periodic spreading and contraction, driven by variations in surface tension.^12^ For both mercury and gallium-based systems, these processes originate from reversible formation and dissolution of a surface salt or oxide layer, such as mercury sulfate for the mercury beating heart^27^ or Ga_2_O_3_ for gallium-based droplet oscillators.^28^ For Hg droplet oscillators, the surface tension is coupled to a surface contact with an electrode (*e.g.*, a nail) and periodic Hg_2_SO_4_ formation/dissolution, resulting in cyclic spreading/contraction and oscillatory behavior. For gallium-based droplet oscillators in alkaline electrolytes (aqueous NaOH) under a constant applied voltage, a three-step mechanism has been proposed.^28^ (i) When the gallium-based droplet contacts the anode, electrochemical oxidation leads to the formation of a self-limiting Ga_2_O_3_ layer,

1 2Ga + 6OH^−^ → Ga_2_O_3_ + 6e^−^ + 3H_2_O

In the presence of oxidizing potentials, the surface tension of the gallium-based droplet is governed by the potential dependent balance between the Ga_2_O_3_ formation and its dissolution in the alkaline solution. Without oxide, the surface tension is around 500 mN m^−1^, whereas when an oxide layer is sustained on the interface, surface tension drops substantially to as low as 50 mN m^−1^.^13^ This leads to the spreading of the drop and the consequent displacement of its center of mass away from the anode. (ii) Dissolution of the Ga_2_O_3_ layer,

2 Ga_2_O_3_ + 3H_2_O + 2OH^−^ → 2[Ga(OH)_4_]^−^

by the NaOH (1 M) electrolyte solution restores the high surface tension and consequencing spherical shape of the liquid-metal droplet. (iii) When surface tension is restored to its initial high value for an oxide-free surface, the droplet returns to the anode due to a capillary-gravity^28^ or Marangoni force induced by electrowetting,^29^ after which the cycle starts again.

Although this model successfully captures the main features of the oscillatory behavior, it is based predominantly on optical observations of droplet shape and motion. Consequently, several aspects of the electrochemical–mechanical coupling remain unclear, including the temporal relationship between oxide formation and droplet motion, the role of charge-transfer processes during different stages of the oscillation cycle, and the origin of the forces responsible for droplet transport. Direct experimental evidence linking the electrochemical response to the mechanical motion of the droplet remains scarce.

Here, we introduce an experimental platform that enables a synchronized optical and electrochemical characterization of self-sustained GaIn droplet oscillators. We controlled the electrochemical potential while simultaneously measuring the time dependence of the current, *I*(*t*), and by optical means the GaIn droplet dynamics, including that of the surrounding electrolyte. This approach, combined with small (2 cm) dimensions of the electrochemical cell, enabled us to study the stable self-oscillations of the GaIn droplets under well-controlled electrochemical conditions (at voltage *U* ≤ 3 V), while minimizing unwanted side reactions, for prolonged periods of time (up to one hour until we stopped the experiment). By correlating electrical signals with the droplet dynamics, the motion of the surface tracers and the flow patterns in the electrolyte solution, we reveal several previously unreported features of the oscillation cycle: (1) a two-step displacement process during droplet separation from the electrode, (2) the dynamic role of the floating Ga_2_O_3_ layer,^30^ and (3) a rapid reversal of the interfacial flow. Our results provide a more comprehensive understanding of electrochemical-mechanical driving mechanisms in liquid-metal droplet oscillators, which is important for future studies on their behavior or potential applications.

## 2. Materials and methods

### 2.1. Platform design

Fig. 1 shows a schematic illustration and photographs of the unconfined three-dimensional cell made from a 3D-printed container made of VeroClear (below in Section 3.3, we also discuss results from a confined, Hele-Shaw configuration). Transparent glass windows form the sides and bottom of the container, allowing for clear visualization of the droplet. The top of the cell is open to the surrounding air. We used two synchronized cameras to image the droplets from above the cell and from the side. The container was bonded to the glass slides using polydimethyl siloxane (PDMS), prepared by mixing the liquid base and curing agent at a 10 : 1 (w/w) ratio, followed by curing at 65 °C for 2 h. It is important to mention that in our design the droplets are always in contact with the smooth glass surfaces and not with the relatively rough 3D printed surfaces to minimize friction between the droplet and the container. The working electrode (WE) and counter electrode (CE) were made of graphite which can withstand the corrosive electrolyte solution (2 M aqueous NaOH) and does not alloy with the Ga–In alloy, contrary to most metals. We would like to point out that water electrolysis is only significant at high voltages of >3 V in our system (see the Videos S3 and S4), significantly higher than the expected 1.23 V under standard electrochemical conditions,^31^ due to choice of electrolyte and graphite electrode combination as described in ref. 32 (where electrolysis of water becomes important for >2.3 V for 5 M NaOH aqueous electrolyte and graphite electrodes). To ensure the electric field is high enough to induce oscillations, we kept the distance between WE and CE at 2 cm.

**Fig. 1:**
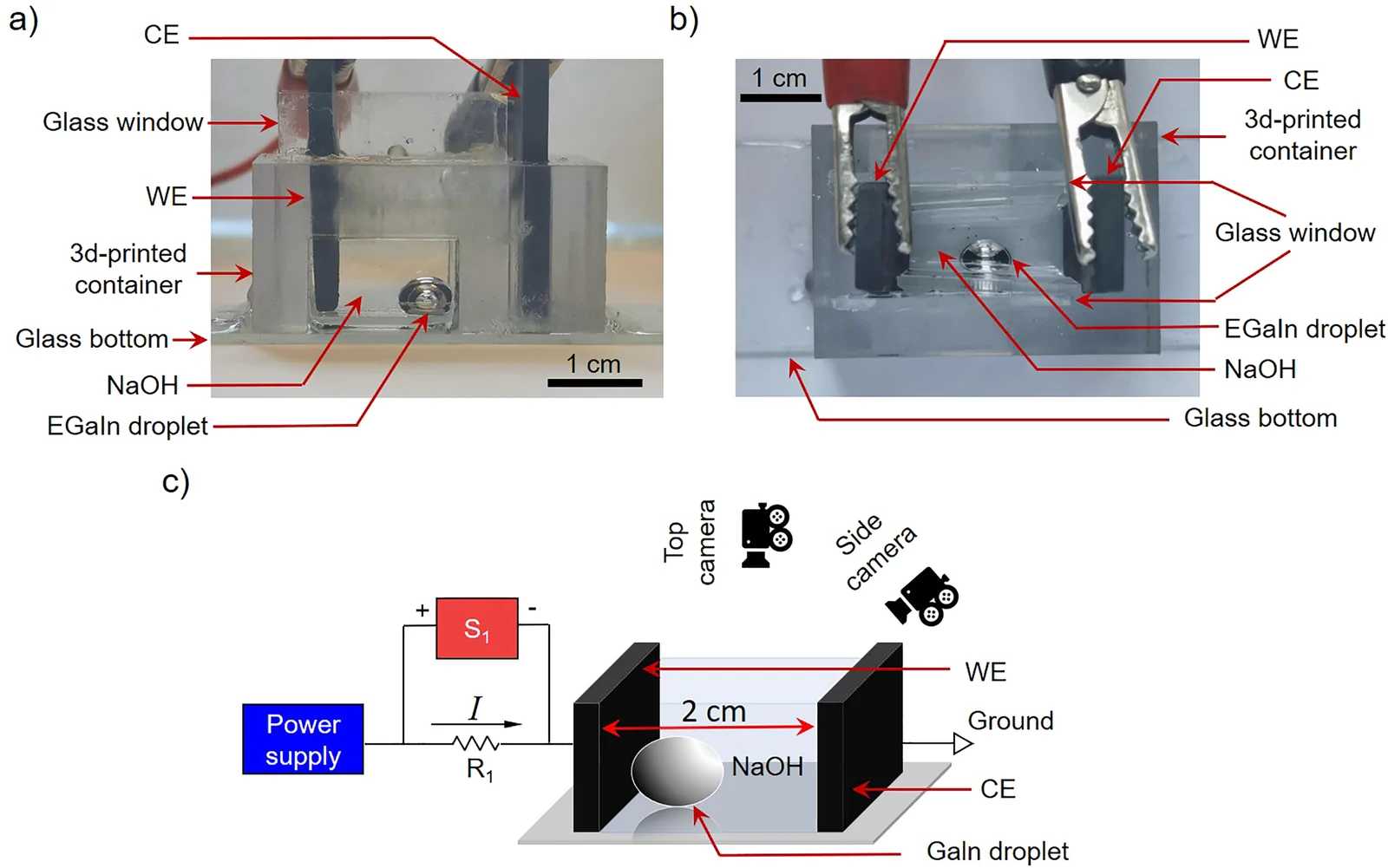
Schematic of the GaIn droplet oscillator device. Photograph of the (a) side-view and of the (b) top-view of the device. (c) Simplified circuit diagram of the device as explained in the text to highlight the electronic circuit where WE is the working electrode, CE is the counter electrode, *R*_1_ is the resistor connected to the droplet system, *I* is the current passing through the whole system, *S*_1_ is the scope to record the voltage across *R*_1_.

In our experiments, we applied a voltage *U* across the WE and CE for a period of time *t*, while recording the droplet movements and surface motion from the top and the side whilst measuring the potential difference across the resistor *R*_1_ (*S*_1_ in Fig. 1c) to obtain the current *I* (in Fig. 1c) through the GaIn droplet oscillator system. To minimize the potential drop across *R*_1_, we used a resistor with a small resistance of 0.994 Ω, which is much lower than the resistance of GaIn droplet oscillator device (see Section 2.3). The electrical response and recorded videos were synchronized by a light emitting diode (LED). The droplet movements were recorded by recording videos at 240 frames/s and the movement of the droplets were tracked by self-developed python code to follow the movement (or distance) of the edge of the droplet with respect to the WE. To visualize the flow around the oscillating droplet, we also used a cell with a confined Hele–Shaw geometry. The inner volume of this cell had a thickness of 3 mm, a distance between electrodes of 2 cm (matching the unconfined three-dimensional cell) and a height of 26 mm. It is also open on the top to surrounding air. It is made of the same glass plates glued with the same PDMS as the unconfined three-dimensional cell.

### 2.2. Chemicals and materials

The eutectic gallium–indium alloy (75.5 wt% gallium and 24.5 wt% indium), EGaIn, was purchased from Sigma Aldrich and used as the liquid-metal droplet as received. NaOH pellets from Sigma Aldrich and ultraclean water from MilliQ were mixed to prepare 2 M NaOH as the electrolyte solution. PDMS was made from SYLGARD™ 184 silicone elastomer kit, purchased from the Dow chemical company. The graphite (ESYNTH018-1EA) was purchased from Sigma Aldrich and cut into pieces to be used as the electrodes. Glass slides (pre-cut microscope slides) were purchased from Epredia.

### 2.3. Electrical circuit

An analog discovery 2 (AD2) data acquisition system and breadboard were used to build the supporting circuit (Fig. 1c) to apply the voltage between WE and CE and to record voltages over a small current sensing resistor *R*_1_. An instrumentation amplifier (INA 126P) was utilized to amplify the voltage over *R*_1_ for the determination of the current flowing through the whole circuit (see SI). The resistance of *R*_1_ is determined by using a four-point measurement with Keysight 34461A, yielding a value of 0.994 Ω. By simply dividing the applied voltage by the recorded *I* (in Fig. 2a), we obtained the resistance of the whole GaIn droplet oscillator system which was from ∼153 Ω to 333 Ω. Hence, *R*_1_ = 0.994 Ω was small enough to ensure it did not affect the effective applied voltage significantly.

**Fig. 2:**
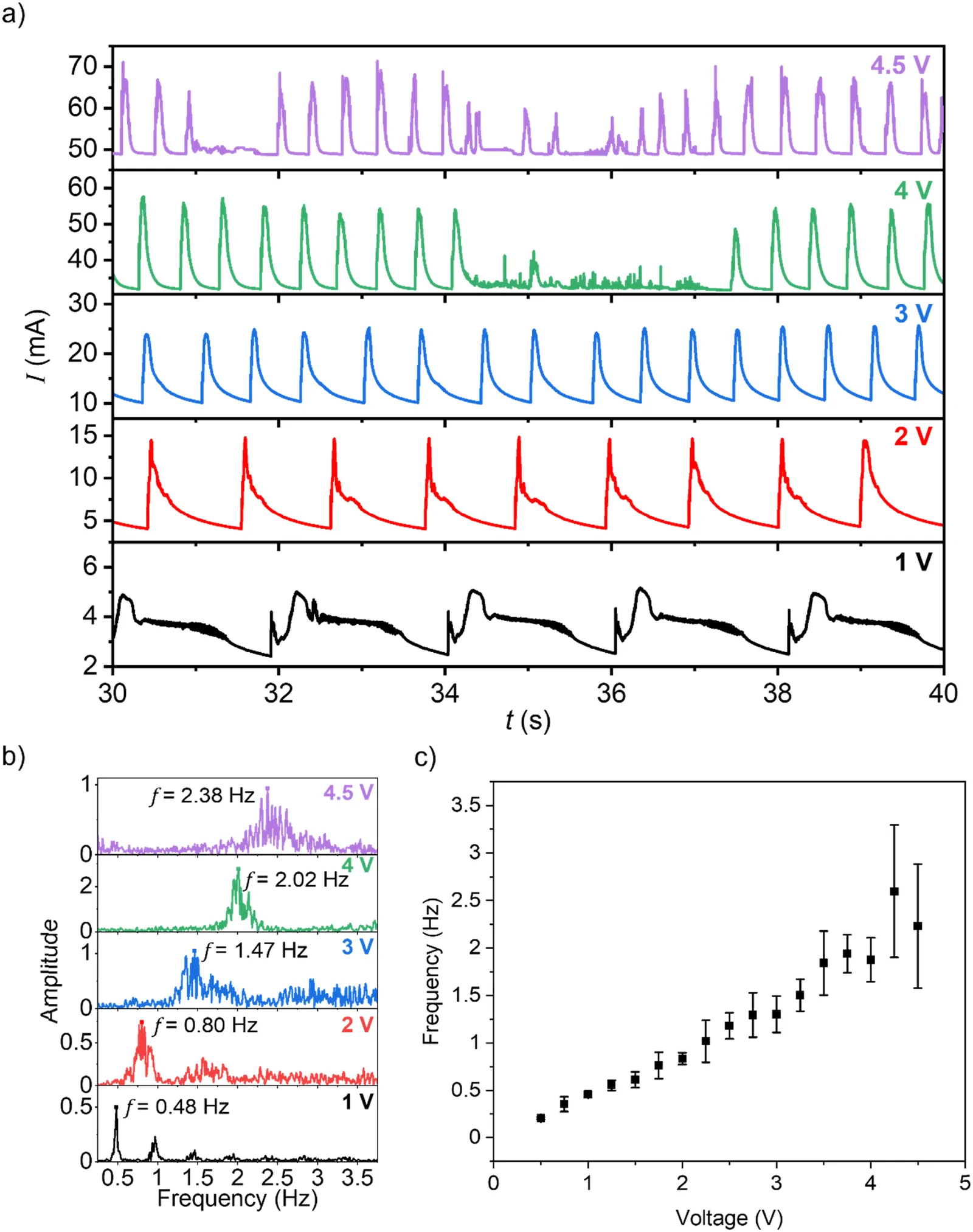
Oscillatory behavior for 100 µL GaIn droplets in 2 M NaOH. (a) Typical *I*(*t*) curves for voltage *U* = 1 V, 2 V, 3 V, 4 V and 4.5 V, from bottom to top; (b) FFT results of these *I*(*t*) curves for *U* = 1 V, 2 V, 3 V, 4 V and 4.5 V; (c) frequency dependence on the voltage. The error bars are the standard deviations determined from 3 experiments.

### 2.4. 3D printing of cells and setups

The container was fabricated using a Stratasys Objet 30 Pro printer with Veroclear as the printing material. Three cut microscope slides were glued on the bottom and inner sides of the 3D-printed cell with PDMS (Fig. 1) to ensure low roughness and to improve visual transparency for imaging. The dimensions of the 3D-printing container were as follows: the width was 12 mm, the height was 15 mm, and the length was 27 mm. The distance between the two graphite electrodes was 20 mm, two graphite pieces (12 mm × 3.5 mm × 30 mm) were placed at the two ends as the electrodes.

### 2.5. Imaging

Two Go pro cameras (model hero 11 black) were used in combination with stereoscopes (magnification 4.5× was used for the recording) to record videos at 240 frames per second with 2.7k video resolution from the top and the side. The Gopro cameras were mounted on the stereoscopes using a custom 3D printed camera adapter. A LED was used to synchronize the motion of the GaIn droplet with the time dependence of the current as follows. By controlling AD2 to send the pulses to the LED light and applying the DC voltage to the droplet oscillator at the same time, the turning on of the LED in the videos can be synchronized to *t* = 0 for the *I*(*t*) data. For the videos from Gopro, Python codes were used to track the movement of the GaIn droplet.

### 2.6. Flow visualization

We visualized interfacial flow motion in both the unconfined three-dimensional cell and the confined Hele–Shaw cell. In the unconfined cell we visualized the surface flow of the GaIn droplet using commercial pink craft glitter (Craft Sensations, The Netherlands). These adsorb at the droplet surface, thus enabling interfacial flow observation. In the Hele–Shaw geometry, we used 20 µm-diameter colorless polystyrene beads (Dynoseed TS-20) in the solution and a Photron high speed camera at 500 fps imaging frequency with a long-distance microscope for recording the images at a spatial resolution of 12 µm per pixel. The images were analyzed by a self-developed particle tracking velocimetry (PTV) algorithm. For both cases of flow visualization, the ability of the tracer particles to follow the flow field with enough precision was assessed using the Stokes number, St = *τ*_p_/*τ*_f_, where *τ*_p_ is the particle response time and *τ*_f_ is the characteristic flow time scale. This criterion is commonly used because particle–fluid slip limits the accuracy of optical velocimetry methods such as particle image velocimetry (PIV) or PTV.^33,34^ The Stokes number was evaluated to St < 3 × 10^−2^ for both cases, indicating that particles follow the streamlines well. Although particle motion in a Hele–Shaw cell can be influenced by confinement, the particle diameter was much smaller than the dimension of both GaIn droplet and cell gap, minimizing wall-induced effects and allowing the particles to reliably represent the local flow field.

## 3. Results and discussions

### 3.1. Electrochemical characterization of the GaIn droplet oscillator

We first characterized the oscillatory response of the GaIn droplets as a function of applied voltage (WE *vs.* CE) to identify an operating regime with stable oscillations. We studied a droplet of 100 µL in 2 M aqueous NaOH and applied different voltages *U* from 0.5 V to 4.5 V with 0.25 V increments. We collected three independent datasets for each voltage.

Fig. 2a shows typical *I*(*t*) plots, for 10 s intervals of the oscillatory behavior at *U* = 1, 2, 3, 4, and 4.5 V (see Videos S1–S5). The corresponding full 80 s *I*(*t*) plots for all applied voltages of all three datasets are provided in the SI (see Fig. S2). For *U* ≤ 3 V, the *I*(*t*) oscillations are periodic, while for *U* > 3 V the irregular *I*(*t*) oscillations occurs. Interestingly, for *U* ≤ 1.5 V, two distinct current transients can be resolved within each oscillation cycle (see Fig. 2a, *I*(*t*) at *U* = 1 V and Fig. S3g–i). As shown in the mechanistic analysis below in Section 3.2, these features arise from electrochemical processes occurring on different timescales, including positive charging (step i in Section 3.2) and oxidation (step ii in Section 3.2) which are distinguishable at low voltages (≤1.5 V) but overlap and merge at higher voltages (>1.5 V).

Fast Fourier transform (FFT) analysis was performed to determine the dominant oscillation frequency and its dependence on the applied voltage, rather than to classify the oscillatory dynamics. For each voltage, the characteristic frequency was defined as the frequency corresponding to the maximum FFT amplitude. Although the oscillations become irregular at higher voltages, the dominant spectral peak still provides a useful measure of the characteristic oscillation frequency. Information regarding the periodicity and regularity of the oscillations is instead provided by the phase portrait shown in Fig. 3g and h respectively. Fig. 2b shows the corresponding FFT spectra. Although the oscillations remain highly reproducible at 2 V and 3 V, the FFT peaks are noticeably broader than those observed at 1 V. This broadening indicates temporal fluctuations in the instantaneous oscillation frequency and suggests that the underlying electrochemical dynamics vary slightly from cycle to cycle. For consistency, we defined the frequency *f* at the highest amplitude in FFT spectra as the oscillation frequency. Fig. 2c shows that the oscillation frequency increases with increasing applied voltage, which is consistent with prior work.^27^ From these observations, we conclude that the device enables stable and reproducible oscillatory behavior in the low-voltage regime (0.5 V ≤ *U* ≤ 3 V).

**Fig. 3:**
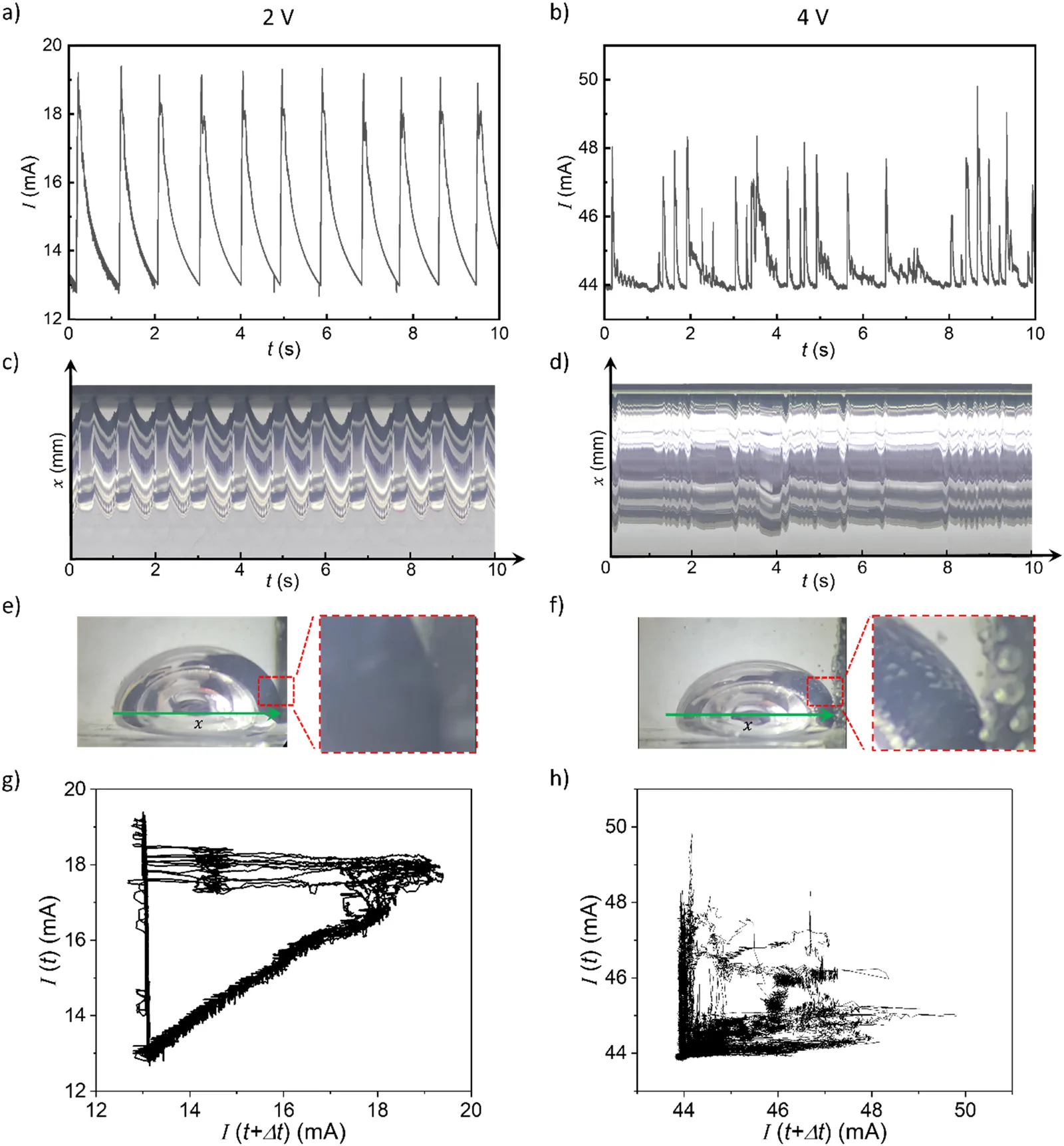
Transition from periodic to irregular oscillations. (a) and (b) *I*(*t*) curves for voltage *U* = 2 V and 4 V, respectively; (c) and (d) kymographs (space–time diagrams) of the oscillations for *U* = 2 V and 4 V, obtained from the image sequences from Videos S2 and S4, respectively; (e) snapshots of videos showing no bubble formation on WE for *U* = 2 V; (f) snapshots of videos showing bubble formation on WE for *U* = 4 V; (g) phase portrait with Δ*t* = 0.0548 s (∼0.05 periodicity) for *U* = 2 V; (h) phase portrait with Δ*t* = 0.0874 s (∼0.05 periodicity) for *U* = 4 V.

Fig. 3 shows a comparison in the oscillatory behavior between the low (2 V) and the high (4 V) potential difference across the cell. The current response at 2 V is highly periodic (Fig. 3a), whereas the oscillations at 4 V exhibit pronounced cycle-to-cycle variability (Fig. 3b). Fig. 3c and d show the corresponding kymographs (space–time diagrams), obtained from the image sequences from Videos S2 and S4, respectively, along the green arrows shown in Fig. 3e and f, respectively. At *U* = 2 V, the pattern in the kymograph is periodic (Fig. 3c) and no gas bubble formation is observed on the graphite electrode (Fig. 3e). In contrast, at *U* = 4 V the kymograph pattern is irregular (Fig. 3d) and gas bubble formation on the graphite electrode is visible (Fig. 3f). For the sake of completion, we analyzed the time-delay phase portraits^35^ of the current response. The embedding delay, Δ*t*, was chosen empirically as 5% of the dominant oscillation period obtained from above FFT results, which provided a well-unfolded phase-space reconstruction while minimizing redundancy between successive coordinates. The phase portrait shown in Fig. 3g for *U* = 2 V converges to a well-defined closed trajectory, characteristic of a stable, self-sustained oscillator. However, the phase portrait shown in Fig. 3h fails to form a closed loop at *U* = 4 V, which indicates the loss of periodicity. We attribute this behavior of unstable oscillations at voltages *V* > 3 V to electrochemical side reactions such as electrolysis. The bubbles formed on the WE disrupt the periodic dynamics. Therefore, all mechanistic investigations presented below focus on the stable oscillatory regime (for 0.5 ≤ *U* ≤ 3 V) where oscillations are not perturbed by the bubble formation on WE. In principle, the stability of the system is limited to the availability of Ga, because Ga continues to dissolve as Ga^3+^ with each cycle (eqn (1) and (2)). Over the course of our experiments, the change in OH^−^ concentration is expected to be negligible. Assuming that a 0.7–5 nm thick GaO_*x*_ layer covering the entire surface of a 100 µL droplet completely dissolves and reforms during each oscillation cycle, the estimated OH^−^ consumption is 4.6 × 10^−9^–3.3 × 10^−8^ mol per cycle, or 7.3 × 10^−8^–9.2 × 10^−6^ mol over the course of 80 s (for frequency range of 0.2–3.5 Hz). Thus, in our experiments with an initial 5.0 × 10^−3^ mol OH^−^ present (volume in our experiments was 2.5 mL), the changes in [NaOH] are <0.2%. To demonstrate that the oscillations are sustained even over a prolonged period of time, we applied *U* = 2 V for 1 h during which the oscillation remained stable (Video S6), though we note that the oscillations were temporarily interrupted when the GaIn droplet adhered to the WE between approximately 900 s and 1011 s which could be caused by an irregularity of the walls or dust particles for example (but not due to a limiting intrinsic property of the system). Photographs taken at *t* = 0 and 3600 s (Fig. S4c and d) show that the solution remained clear and that no graphite particulates were present. These observations indicate that during the timescale of our experiment reported here, consumption of Ga did not hinder the mechanistic investigations.

### 3.2. Driving mechanism of the GaIn droplet oscillator

The voltage-dependent current responses described above reveal electrochemical features that cannot be inferred from optical observations alone. In the Introduction, we have summarized the proposed three-step mechanism involving oxide formation, oxide dissolution, and droplet return to the electrode, but the temporal relationship between these electrochemical processes and the accompanying droplet deformation and motion has remained unclear. In particular, it is unclear how Ga_2_O_3_ formation and dissolution couple to droplet spreading, contraction into spherical shape, Marangoni flow (see below) and droplet motion away from and towards the electrode, during different stages of the oscillation cycle.

Therefore, we simultaneously recorded the *I*(*t*) while imaging a 100 µL GaIn droplet at *U* = 2 V. The image sequences were used to track the left and right droplet boundaries, together with surface motion. Fig. 4 shows a full oscillation period from an arbitrary time *t* = 2.05 s to *t* = 3.05 s. The video showing this oscillation period is available in the SI Video S2. Fig. 4a shows the simultaneous current *I*(*t*) in red and the distance of the left and right extremities of the droplet with respect to the WE in green and black, denoted *d*_L_ and *d*_R_, respectively. In blue, the distance *d*_S_ of a surface-adsorbed polystyrene particle (encircled in Fig. 4b) with respect to the WE is plotted. These distances are indicated in Fig. 4b. We added dye-colored particles, to visualize the surface motion of the droplet. Since bare GaIn has such a high surface tension, we can safely assume that the particles were attached to the Ga_2_O_3_ allowing us to track surface motion. Fig. 4b shows snapshots of the oscillating droplet from a synchronized side and top view. From these data, we identify the following 5 steps in the oscillatory mechanism involving (i) contact of the droplet with WE (contact), (ii) formation of Ga_2_O_3_ layer (oxidation), (iii) detachment of droplet from the WE (contraction), (iv) movement away from WE (separation), and (v) continuous-electrowetting (CEW) driven movement back toward WE (electrowetting). These steps are labeled accordingly in Fig. 4a.

**Fig. 4:**
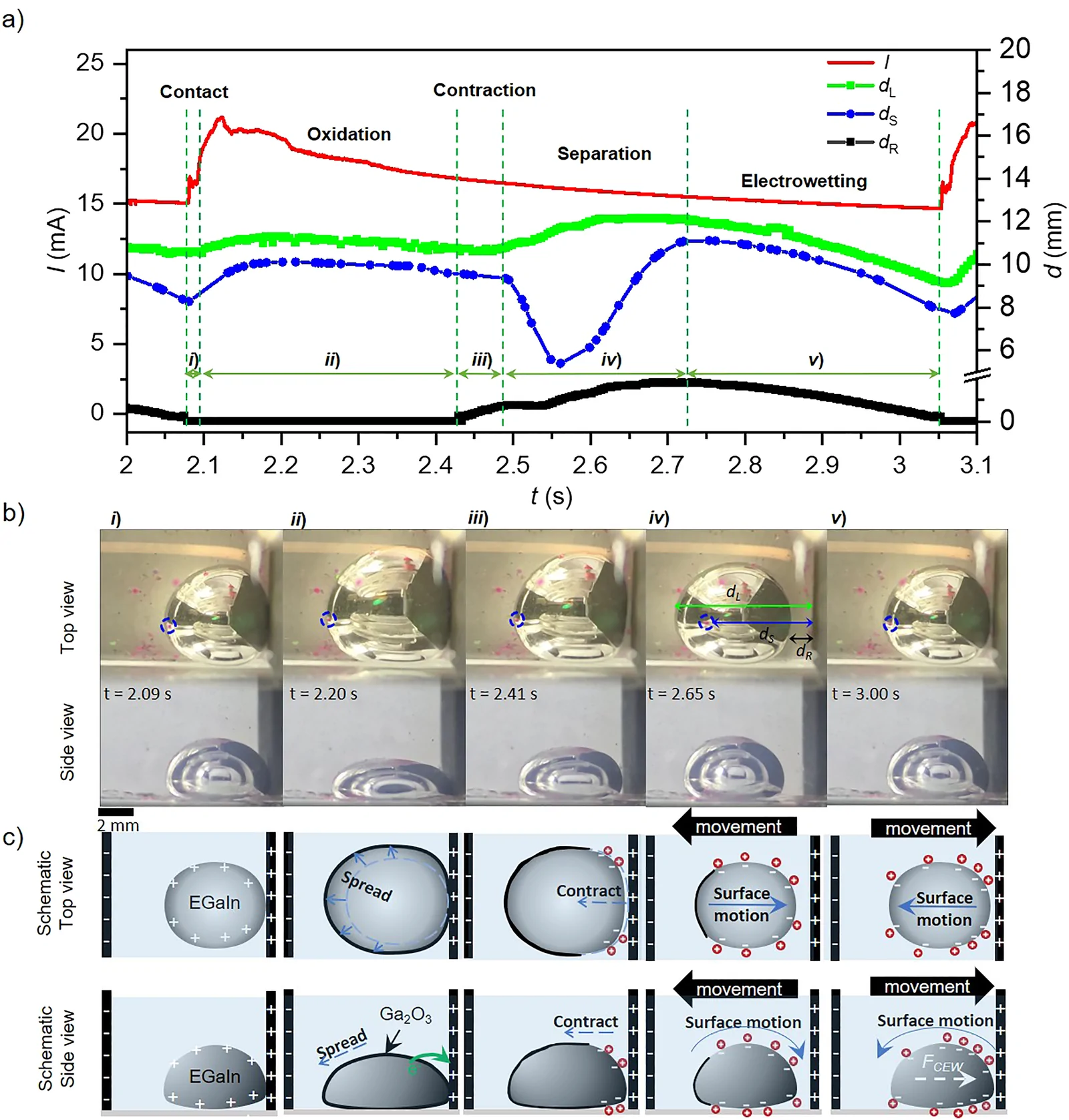
The different steps involved in the mechanism of oscillation of the GaIn droplet oscillator. (a) *I*(*t*) curve (red, top) produced by the GaIn droplet oscillator for voltage *U* = 2 V and surface motion *d*_S_ (blue, middle), motion of the left side *d*_L_ (green, top) and right side *d*_R_ (black, bottom) of droplet as a function of *t*. The green dashed lines indicate the steps (i) to (v) represented in the corresponding photos (b) and schematic illustrations (c) as explained in the main text.

#### Step (i) droplet–electrode contact and positive charging

At *t* = 2.07 s, the GaIn droplet comes to contact with the WE (Fig. 4b(i)), resulting in a sharp peak in the *I*(*t*) curve (Fig. 4a(i)). This increase in the current is consistent with the rapid change in the electric charge of the droplet (as schematically drawn in Fig. 4c(i)).

#### Step (ii) oxide growth and droplet spreading

In Fig. 4b(ii) on the top view it is apparent that the footprint of the droplet has increased, indicating spreading on the substrate under the effect of gravity. This suggests a substantially lower surface tension compared to that before contact with the electrode. We attribute this to the formation of the Ga_2_O_3_ layer on the surface of the droplet. Interestingly, the *I*(*t*) curve increases and then decreases. This behavior can be explained by the self-limiting nature of Ga_2_O_3_ formation,^36^ whereby the growing oxide layer progressively inhibits further charge transfer once the surface becomes fully covered (around *t* = 2.22 s). Alternatively, the decrease can be due to a reduction in the contact area between the drop and the WE. During this process, the droplet continues to deform and flatten due to the low surface tension of the Ga_2_O_3_ under the influence of gravity (Fig. 4b(ii)), effectively pushing the droplet's center of mass away from the WE. This is apparent in Fig. 4a by the increase of *d*_L_, while *d*_R_ remains zero. By analyzing droplet contours using Young–Laplace fits, we obtained Bond numbers of approximately Bo ≈ 2.2 and Bo ≈ 1.0 for the unoxidized and oxidized states, corresponding to surface tensions of about 0.48 and 0.21 N m^−1^, respectively. These values yield inertiocapillary and viscocapillary timescales of order 10 ms and 10 µs, respectively. These time scales are both much smaller than the oscillation period (∼1 s), indicating that the oscillation frequency is primarily driven by voltage-dependent electrochemical processes.

#### Step (iii) droplet detachment and contraction

At *t* = 2.43 s, the droplet separates from the WE characterized by an increase in *d*_R_. This event produces no obvious feature in the current trace, highlighting the importance of combining electrical and optical measurements. During the detachment of the GaIn droplet from the WE (step iii to v), the current decays exponentially with a characteristic decay time of 0.552 s (see Fig. S5). This back ground current may involve interfacial charging of the graphite electrodes or ionic currents across the electrolyte solution for example. The separation starts with slowing down of the thickening of the oxide layer since it is a self-limiting process. As this oxidation process slows down, the dissolution of the oxide layer by the NaOH becomes increasingly dominant to a point where dissolution of the oxide layer restores the high surface tension of the liquid-metal droplet, causing it to contract. This contraction is reflected by the decrease in *d*_L_ and the increase in *d*_R_, accompanied by a progressive recovery of a nearly spherical droplet shape (Fig. 4b(iii), top view) while transitioning from step (iii) to (iv). Note how after detachment at *t* = 2.41 s, the droplet still holds the approximately flat edge on its right side. This is another indication of the presence of the oxide layer since it is well-known the gallium oxide skin gives the GaIn surface rigidity.^37^

#### Step (iv) inertial movement and competing surface flows

At *t* = 2.48 s, following detachment, the droplet continues to move away from the WE under inertia, which is the result of the contraction of the droplet from the asymmetrical rectangular shape to the symmetrical spherical-cap shape. Unlike in step (iii), this time *d*_L_ and *d*_R_ increase simultaneously indicating translation of the entire droplet. Accompanying the movement of the droplet away from the WE, the adsorbed particle moves closer to the WE with faster speed. This is apparent from Fig. 4a by the decrease of *d*_S_. We attribute this to a combination of the surface shear induced by the asymmetrical contraction and the consequent bulk movement of the GaIn droplet. Note that around *t* = 2.55 s, while the droplet is still moving away from the electrode under the effect of inertia, *d*_S_ starts increasing faster than *d*_L_. This indicates that the surface flow of the droplet reversed, interfacial flow velocity is from the right side of the droplet towards its left side. This is due to the Marangoni flow induced by the CEW effect causing an interfacial flow and a pressure difference across the droplet, which starts slowing down the speed of the droplet moving away from the electrode and subsequently propelling it towards the WE. The separation phase ends when the droplet stops and reverses its direction of movement and the adsorbed particle starts moving together with the droplet. The appearance of this interfacial flow is further confirmed in Section 3.3 in the Hele–Shaw geometry.

#### Step (v) electrowetting-driven return to the electrode

At *t* = 2.73 s, the droplet reverses direction and begins moving towards the working electrode (Fig. 4a(v)). At this point, its movement is driven by the electrowetting-induced Marangoni force. The applied electric field polarizes the droplet and due to the potential difference between its left and right side, creates a surface-tension gradient across the liquid-metal/electrolyte interface, generating a net force toward the electrode. As the droplet approaches the electrode, electrical contact is re-established after which the whole process can start over again from step (i).

### 3.3. Flow visualization of the competing Marangoni flows

The synchronized measurements presented in Section 3.2 suggest that: (1) the droplet's center of mass is pushed away from the electrode by its spreading and subsequent asymmetrical contraction due to its inertia. (2) Afterwards, the droplet is pulled back towards the electrode by a Marangoni flow due to continuous electrowetting. To directly visualize the fluid motion surrounding the oscillating droplet and verify this hypothesis, we performed particle-tracking velocimetry (PTV) measurements in the Hele–Shaw cell described in Section 2.6.

Fig. 5 shows snapshots of the oscillating droplet from Video S7 together with the corresponding velocity fields and streamlines. The working electrode appears as a black line on the right side of the images. In step (i), at *t* = 0.05 s, the droplet comes into contact with the WE. In step (ii), at *t* = 0.16 s the droplet is spreading which causes its center of mass to move away from the WE towards the left, due to the decrease in surface tension attributed to the oxide layer formation. In step (iii), at *t* = 1.77 s the droplet has detached from WE and is separating from it. We attribute this to the left side of the droplet (after the spreading due to the oxide layer formation) wetting the surrounding substrates more than the right, effectively causing an asymmetrical contraction once the surface tension returns to its original value of around 500 mN m^−1^. Note that the streamlines originate from the left side of the droplet with an angle close to perpendicular to its interface. They go around the droplet and join back into it on the right side, also at angles close to perpendicular to the interface. This indicates that fluid motion is caused by the shape change and movement of GaIn droplet due to asymmetrical contraction. In step (iv), at *t* = 1.87 s, the droplet has nearly stopped moving, as its contraction-induced motion is more strongly damped by the additional confinement and friction from the cell walls, compared to the unconfined motion in the three-dimensional cell. At *t* = 2.03 s, a pronounced interfacial flow develops while the droplet remains essentially stationary. The appearance of this flow, prior to the motion of the droplet towards the WE, provides direct evidence that interfacial stresses are being established before the droplet begins to move. As the interfacial flow strengthens, the droplet starts accelerating back to the WE. At *t* = 2.25 s, when the droplet is moving towards the WE, the velocity field exhibits that streamlines are aligned with the surface and they detach from the droplet at its left side. Moreover, the largest velocity values are found very close to the surface. These are characteristics of a flow driven by the Marangoni effect. The flow direction is consistent with the electrically induced surface-tension gradient proposed in step (v) of the unconfined three-dimensional cell.

**Fig. 5:**
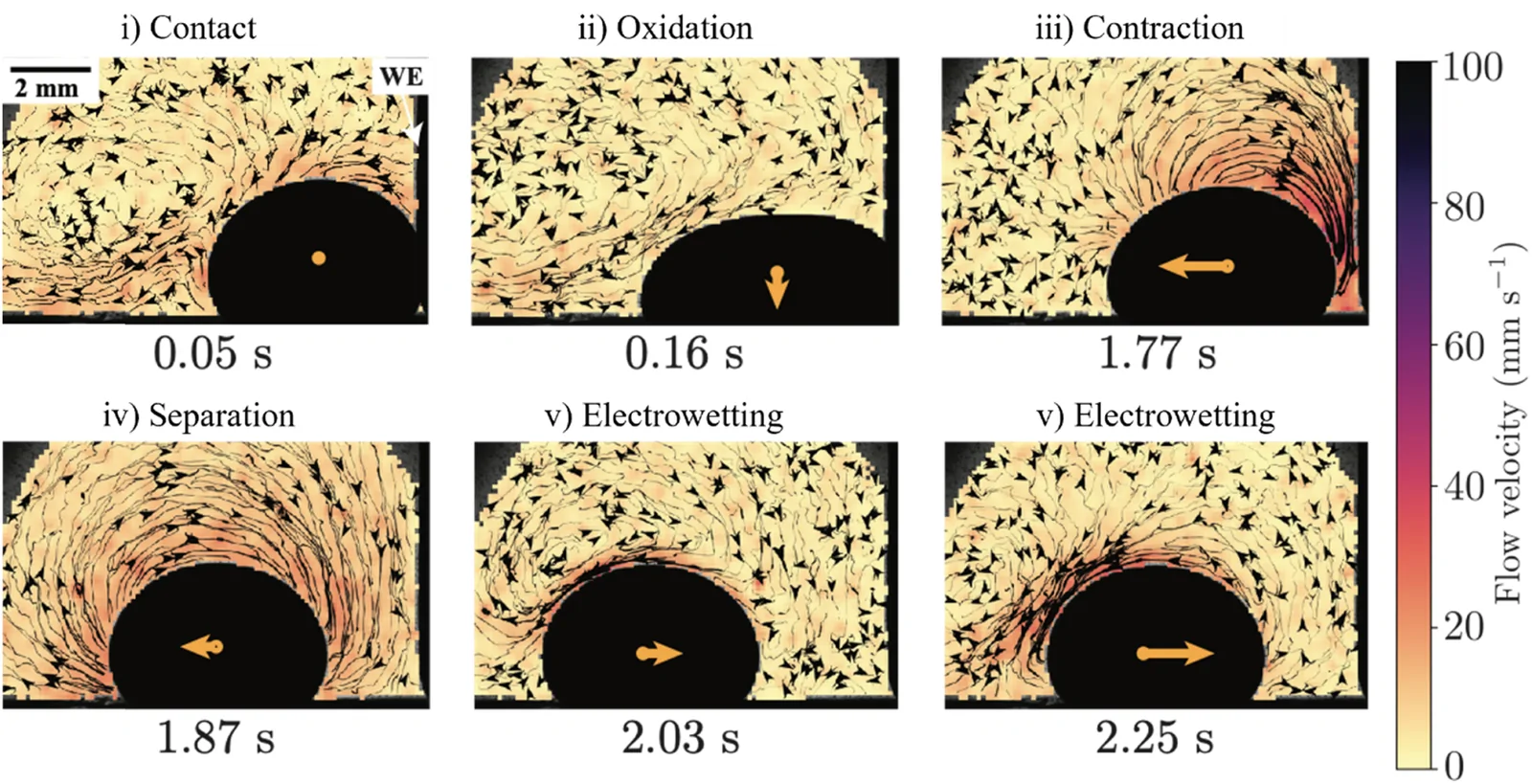
Snapshots of flow visualization of the oscillating GaIn droplet in the confined Hele–Shaw cell, for voltage *U* = 2 V. Velocity field is calculated from particle tracking velocimetry and the local velocity magnitude is indicated as a color map. The working electrode (WE) is located at the right edge of the images. The black lines with black arrows surrounding the GaIn droplet are calculated streamlines of the flow. The orange arrows at the center of the GaIn droplet indicate the direction of its movement, with arrow lengths qualitatively representing the relative droplet speed.

We note that the oscillation period in the Hele–Shaw cell *T*_HS_ ≈ 2.45 s is larger than that observed in the standard cell configuration *T*_3D_ ≈ 0.9 s. This difference is likely due to increased confinement and friction between the droplet and the cell walls. Nevertheless, the same sequence of events is observed. The flow visualization in the Hele–Shaw configuration therefore provides independent validation of the electrochemical–mechanical driving mechanism proposed in Section 3.2 that results in self-oscillations.

## 4. Conclusions

In summary, we developed an experimental platform that enables simultaneous electrochemical and optical characterization of self-sustained GaIn liquid-metal droplet oscillators under well-controlled electrochemical conditions while minimizing electrolysis of water and avoiding electrode degradation. By simultaneously recording the motion of the droplets from a top and a side view, while measuring the currents combined with flow visualization (in the Hele–Shaw configuration), we were able to study the mechanism behind the volage-driven self-oscillations and identified five distinct steps in the oscillation cycle: (i) droplet-electrode contact and positive charging, (ii) spreading of droplet due to oxide formation, (iii) droplet detachment and contraction due to oxide dissolution, (iv) inertial movement away from the electrode due to asymmetrical contraction and (v) Marangoni-flow driven movement back to electrode due to continuous electrowetting.

More broadly, this work demonstrates the value of combining synchronized electrochemical and optical measurements for studying an active oscillatory system. Whereas previous investigations of liquid-metal droplet oscillators have relied predominantly on visual observations, the simultaneous acquisition of current responses provides direct access to how electrochemical processes (oxide formation and dissolution) couple to surface motion and, in turn, movement of the droplets. The mechanistic insights obtained here contribute to a more complete understanding of the coupled electrochemical–mechanical driving mechanism in suspended liquid-metal droplets and establish an experimental framework for future studies of oscillatory liquid-metal systems and potentially other systems.

## Conflicts of interest

There are no conflicts to declare.

## Supplementary Material

SM-OLF-D6SM00708B-s001

SM-OLF-D6SM00708B-s002

SM-OLF-D6SM00708B-s003

SM-OLF-D6SM00708B-s004

SM-OLF-D6SM00708B-s005

SM-OLF-D6SM00708B-s006

SM-OLF-D6SM00708B-s007

SM-OLF-D6SM00708B-s008

SM-OLF-D6SM00708B-s009

## Data Availability

The code to control the applied voltage and to record the signal is based on MATLAB, and the code for tracking the movement of the GaIn droplet is based on Python. These codes and all data are available upon request from the corresponding author. Supplementary information (SI) is available. See DOI: https://doi.org/10.1039/d6sm00708b.
