# Supplementary material for: Self-sustained GaIn droplet oscillations by coupled electrochemical and mechanical driving forces
Source: Soft Matter. 2026 Sep 29. Online ahead of print. doi: 10.1039/d6sm00708b (PMC13656080; doi:10.1039/d6sm00708b)
Supplement: Supplementary file 1 [file SM-OLF-D6SM00708B-s001.pdf]

Supplementary Information (SI) for Soft Matter.
This journal is © The Royal Society of Chemistry

Self-Sustained GaIn Droplet Oscillations by Coupled Electrochemical and Mechanical Driving Forces

Yi Li,[1] David Paulovics,[2] Mathieu Odijk,[3] Javier Rodríguez Rodríguez,[4] Ivana Qianqi Lin,[1] Detlef Lohse,[2,5] and Christian A. Nijhuis[1,]*

1. Hybrid Materials for Opto-Electronics Group, Department of Molecules and Materials, MESA+ Institute for Nanotechnology and Center for Brain-Inspired Nano Systems, Faculty of Science and Technology, University of Twente, P.O. Box 2017, 7500 AE Enschede, The Netherlands
2. Physics of Fluids Group, Max Planck Center for Complex Fluid Dynamics, and J.M. Burgers Centre for Fluid Dynamics, University of Twente, P.O. Box 217, 7500AE Enschede, The Netherlands;
3. Integrate Devices and Systems Group, MESA+ Institute, University of Twente, Hallenweg 15, 7522 NH Enschede, the Netherlands
4. Department of Thermal and Fluids Engineering, "Gregorio Millán Barbany" Institute, Universidad Carlos III de Madrid, Spain
5. Max Planck Institute for Dynamics and Self-Organization, Am Faßberg 17, 37077 Göttingen, Germany.

*Author to whom correspondence should be addressed: c.a.nijhuis@utwente.nl

1. Details about the setup.

Figure S1a demonstrates the full diagram of the electrical circuit, where we applied an instrumental amplifier before record the signal from the R_1 because the voltage flow to R_1 is very low to avoid R_1 influence the voltage go through the droplet system. VP+ and VP- are both power supply channel in electrical instrument, Analog Discovery 2 (AD2). VP+ is the power supply for the main circuit while VP- is the power supply for the LED light for synchronizing the electrical data and image. Via controlling AD2 by code, VP+ and VP- can give the potential at the same time which allows us to synchronize the turning on of the LED in the videos with the $t = 0$ for the $I(t)$ data to align the electrical signals and droplet motion. We used high resolution mode of AD2 where the voltage range of the AD2 is from -2.5 V to 2.5 V and added an offset in the output signal by applying an offset voltage (W_1) to fit the range. R_G was used to adjust the amplification of the amplifier, where for U= 0.5 V – 1 V, R_G = 324 Ω, offset voltage = 2 V; U= 1.25 V – 3 V, R_G = 668 Ω, offset voltage = 3 V; U= 3 V – 3.5 V, R_G = 806 Ω, offset voltage = 3 V; U= 3.75 V – 4.5 V, R_G = 1461 Ω, offset voltage = 4 V. As shown in figure S1b, the GoPro cameras are mounted on the stereoscopes with 3d-printed camera adapter to help focus. Optical magnification of stereoscopes helps for studying the details on the droplet.

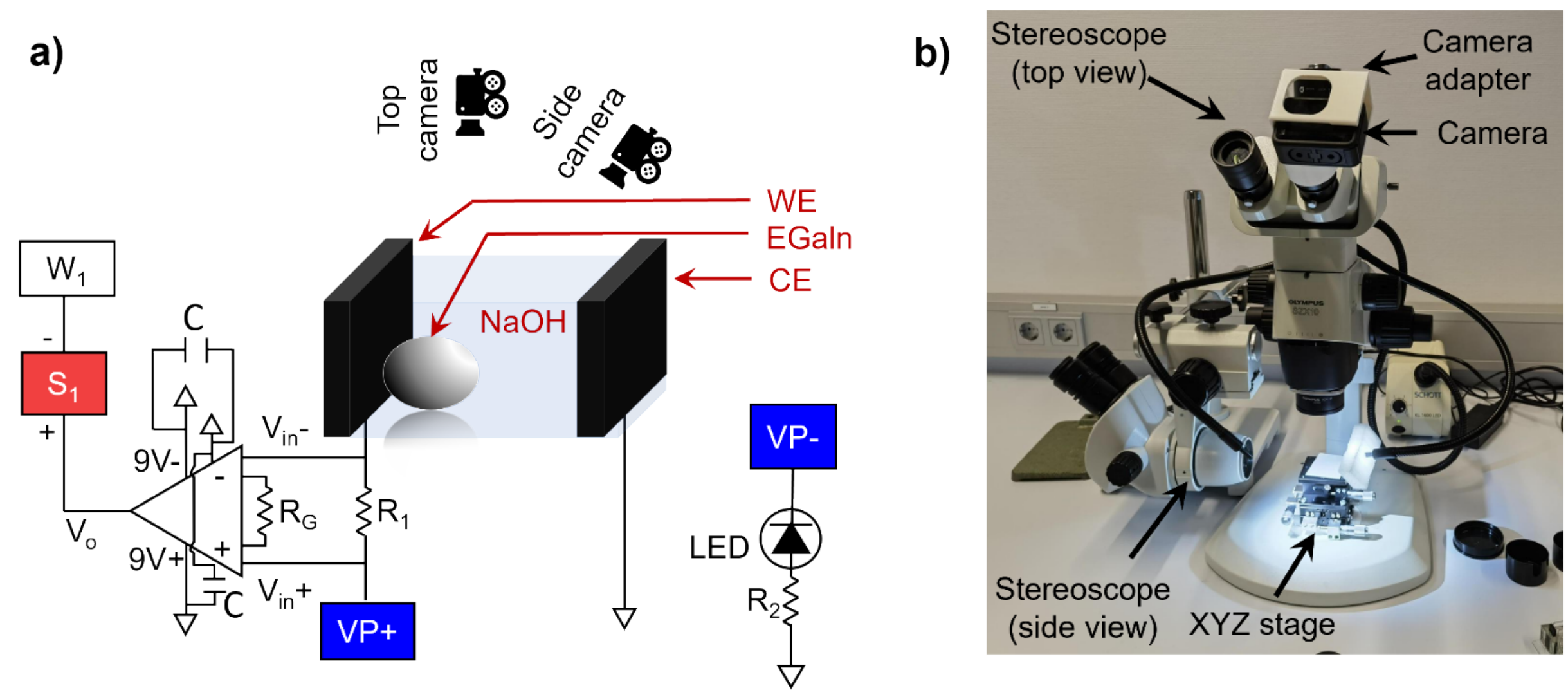

Figure S1. a) Details about the circuit and b) photos of the platform.

2. *I*(*t*) curves for all applied voltages

To demonstrate the reproducibility of this cell, we collected three independent datasets while increasing the applied voltage, as shown in Fig.S2 and Fig.S3. Overall, for 0.5 V ≤ *U* ≤ 3 V, the oscillations are periodic, though there are fluctuations or break in oscillations in some cases, while for *U* > 4 V, the *I*(*t*) oscillations are irregular. As shown in Fig. S3g, h and i, for *U*≤ 1 V, *U*= 1.25 V in experiment 2, and *U*= 1.25 and 1.5 V in experiment 3, two distinct current transients can be resolved within each oscillation cycle. We attribute these features arise from the different timescales between electrical double-layer charging of the droplet when it contacts the working electrode and oxidation process afterwards.

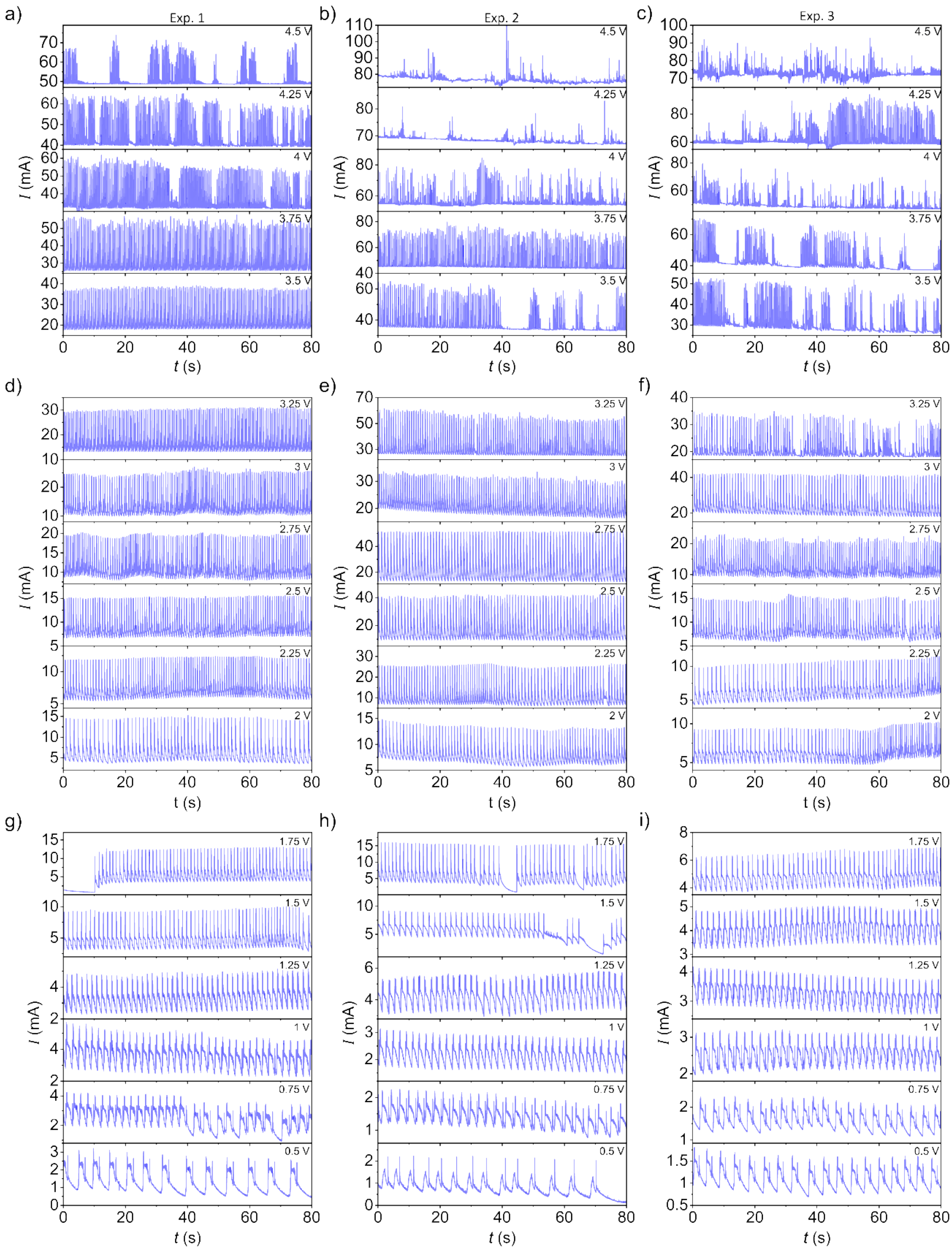

Figure S2. Electrical oscillations for a 100 μL GaIn droplet in 2 Molar NaOH under applied voltage at a), b) and c) $U = 3.5$ V – 4.5 V, d), e) and f) $U = 2$ V – 3.25 V, g), h) and i) $U = 0.5$ V – 1.75 V for three different experiments, respectively.

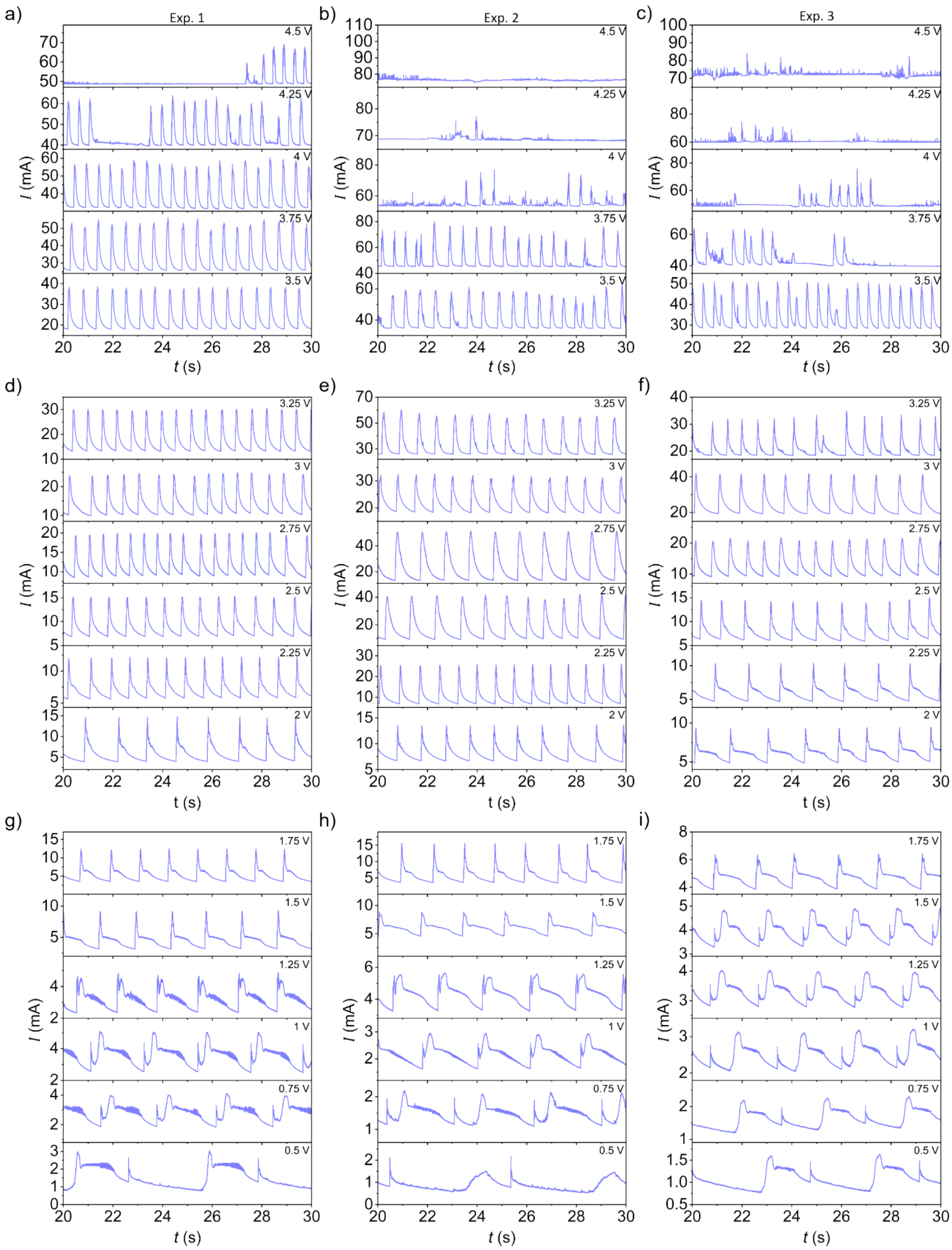

Figure S3. Segments of electrical oscillations for a 100 μL GaIn droplet in 2 Molar NaOH under applied voltage at a), b) and c) U = 3.5 V – 4.5 V, d), e) and f) U = 2 V – 3.25 V, g), h) and i) U = 0.5 V – 1.75 V for three different experiments, respectively.

3. Electrical oscillations for a prolonged period.

To demonstrate that the oscillations are stable over a prolonged period of time, we applied $U = 2$ V for 1 h. Figure S4a shows the overall oscillations remained sustained over 1 hour timescale though there is a current dropping down occurred at around $t = 900$ s to 1011 s, which is due to stuck of the GaIn droplet on the working electrode (see Video S6). In this experiment, H_2 bubbles were generated at the counter electrode as a result of the corresponding reduction reaction as shown in Fig. S4c and S4d. As mentioned in the main text of manuscript, using low applied voltage suppresses the bubbles formation at the working electrode, which is essential to prevent bubble-induced perturbations of the GaIn droplet oscillations.

To quantify the long-term dynamics, we also analyzed the 1 h current-time signal in consecutive 360 s intervals (Fig. S4b). As shown in the figure below, the dominant oscillation frequency increased from approximately 1.1 Hz during the initial stage to approximately 1.6 to 1.8 Hz after about 1500 s. Thereafter, the frequency remained within this range, indicating an initial conditioning period followed by a quasi-steady oscillatory regime. Since the frequency was stable up to 3600 s, we conclude that the total OH^- consumption was still low, toward to the lower end of the calculated consumption (as explained in the main text).

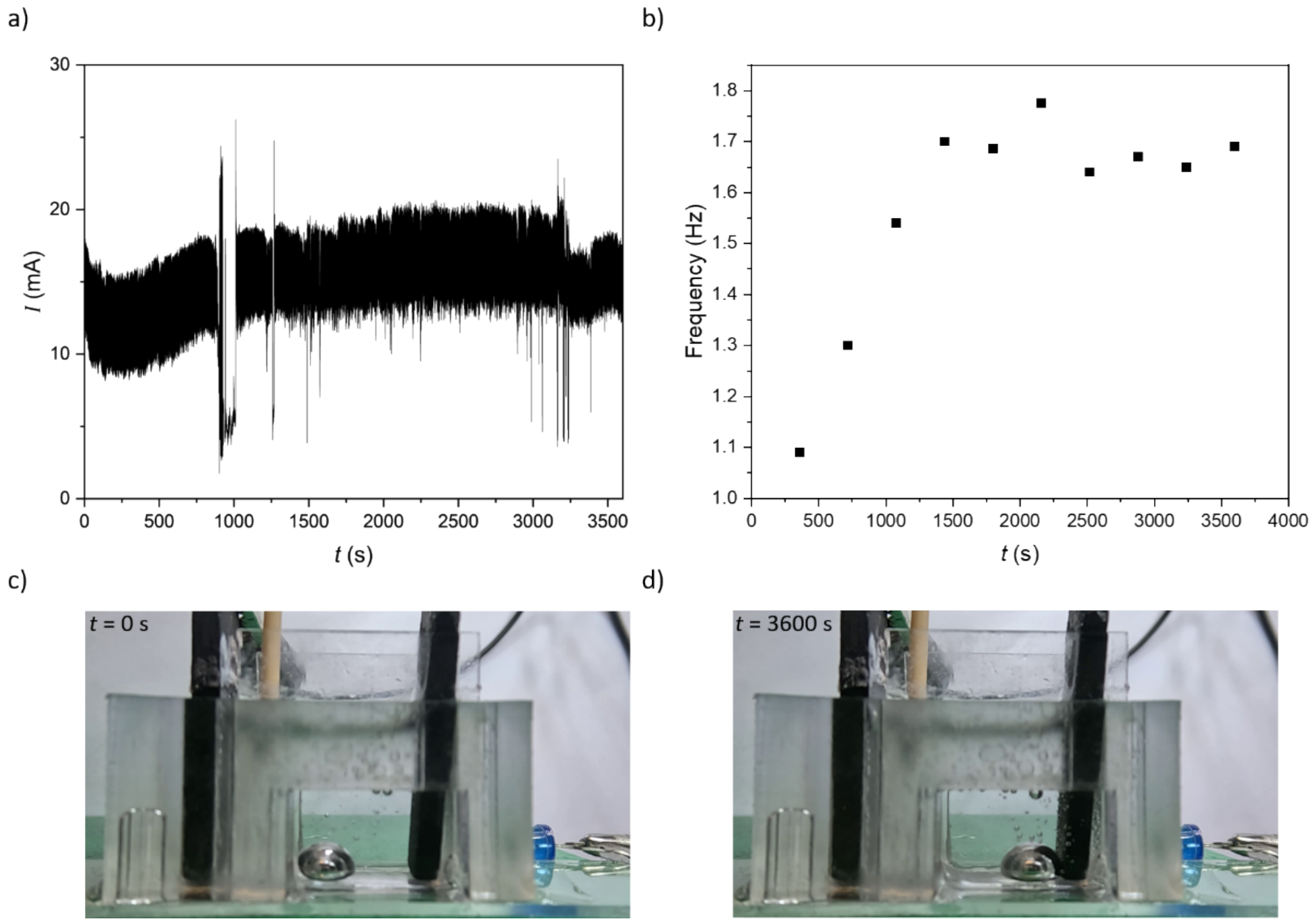

Figure S4. a) Electrical oscillations for 1 h of a 100 μL GaIn droplet in 2 M NaOH at V = 2 V. b) The frequency evolution determined for the 10 consecutive 360 s intervals. c) and d) screenshots of Video S6 at t = 0 and 3600 s, respectively.

4. Exponential fit for *I*(*t*) during detachment of GaIn droplet.

To instigate the property of the electrical behavior during the detachment of GaIn droplet from the working electrode. As shown in the Fig. S5, we isolated the *I*(*t*) curve for *t* from 2.43 s to 3.05 s, corresponding to step iii to v, and fitted it with a first-order exponential decay function:

$$I(t) = I_o + Ae^{(-\frac{t}{\tau})}$$

We obtained a background current I_o = 13.7 mA, pre-exponential factor A = 253 mA, and the characteristic decay time τ = 0.552 s. The fit describes the data with R^2=0.999. Such a decay may be caused by charging effects (double layer formation) and background ionic currents. These effects explain why the current does not change during detachment.

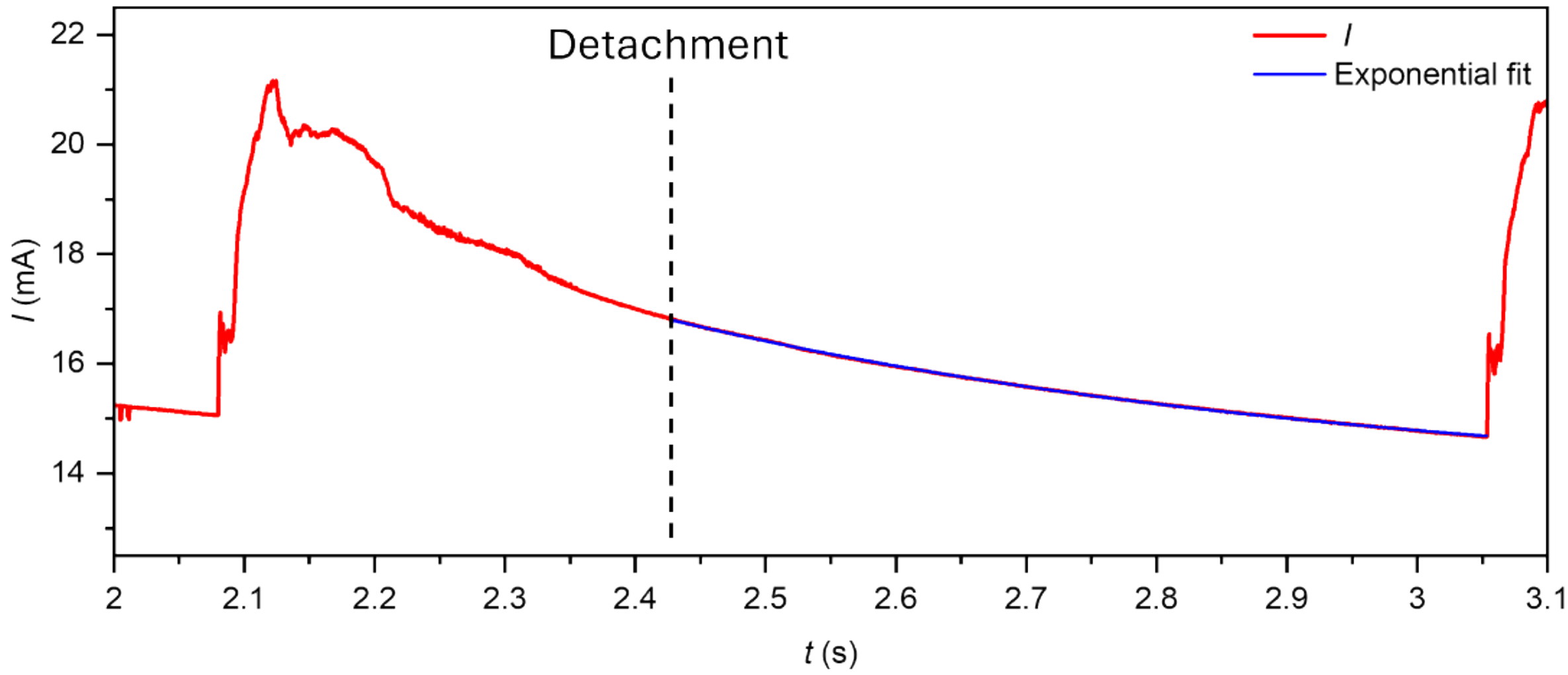

Figure S5. Exponential fit of the current-time data shown in Figure 4. The blue line shows an exponential fit to the current decay after the vertical dashed line indicating the moment when the droplet detaches from the working electrode.
